# Relapse rates in stable obsessive-compulsive disorder after antidepressant discontinuation versus maintenance: A systematic review and meta-analysis

**DOI:** 10.1017/S0033291725101578

**Published:** 2025-08-28

**Authors:** Taro Kishi, Kenji Sakuma, Masakazu Hatano, Shun Hamanaka, Yasufumi Nishii, Nakao Iwata

**Affiliations:** 1 Department of Psychiatry, Fujita Health University School of Medicine, Toyoake, Japan; 2 Department of Pharmacotherapeutics and Informatics, Fujita Health University School of Medicine, Toyoake, Japan

**Keywords:** obsessive–compulsive disorder, antidepressant discontinuation, antidepressant maintenance, relapse, recurrence, prevention, all-cause discontinuation, adverse event-related discontinuation, systematic review, meta-analysis, randomized controlled trial, Evidence-Based Mental Health

## Abstract

**Background:**

The optimal duration for maintaining antidepressant treatment in individuals with obsessive-compulsive disorder (OCD) who achieve symptom stabilization remains unclear.

**Methods:**

This systematic review and pairwise meta-analysis of double-blind randomized placebo-controlled trials (DBRPCTs) compared antidepressant maintenance and antidepressant discontinuation groups in terms of relapse rate at each DBRPCT study endpoint (primary outcome), OCD symptom improvement, all-cause discontinuation, and adverse event-related discontinuation. Furthermore, relapse rates at 4, 8, 12, 16, 20, and 24 weeks were compared between the groups. Risk ratios (RRs) with 95% confidence intervals (CIs) were calculated. The absolute risk reduction (ARR) and number needed to treat to benefit (NNTB) for relapse rates were also estimated.

**Results:**

Nine trials (n = 1084; mean age: 32.8 years; proportion of males: 53.3%) were included. The antidepressant maintenance group had lower relapse rates at each DBRPCT study endpoint (RR [95% CI] = 0.53 [0.42–0.68]; ARR = 21.0%; NNTB = 5) and lower all-cause and adverse event-related discontinuation rates than the antidepressant discontinuation group. The maintenance group also exhibited lower relapse rates at 4 weeks (RR [95% CI] = 0.47 [0.31–0.70]; ARR: not significant; NNTB: not significant), 8 weeks (0.42 [0.31–0.57]; 12.0%; 8), 12 weeks (0.43 [0.32–0.56]; 18.0%; 6), 16 weeks (0.41 [0.32–0.52]; 25.0%; 4), 20 weeks (0.43 [0.34–0.53]; 26.0%; 4), and 24 weeks (0.42 [0.33–0.52]; 27.0%; 4) than the discontinuation group. Moreover, the maintenance group outperformed the discontinuation group regarding OCD symptom improvement.

**Conclusions:**

Individuals with OCD may benefit from continued antidepressant treatment, provided that it is well tolerated.

## Introduction

Obsessive-compulsive disorder (OCD) is a prevalent mental illness, with a lifetime prevalence estimated at approximately 2%–3% (Fontenelle, Mendlowicz, & Versiani, 2006; Hirschtritt, Bloch, & Mathews, 2017; Stein et al., 2019). OCD is characterized by the presence of obsessions and/or compulsions (DSM-5-TR, 2022). Individuals with OCD are treated with pharmacological interventions, such as antidepressants and antipsychotics (Bandelow et al., 2023; Hirschtritt et al., 2017), as well as nonpharmacological therapies, including psychotherapy and neuromodulation (Fineberg et al., 2015; Hirschtritt et al., 2017; Stein et al., 2019). Several treatment guidelines recommend continuing antidepressant treatment for 1–2 years to prevent relapses after remission in individuals with OCD who have been stabilized with antidepressants (Bandelow et al., 2023; de Oliveira et al., 2023; Hirschtritt et al., 2017; Janardhan Reddy, Sundar, Narayanaswamy, & Math, 2017; Koran & Simpson, 2013; NICE, 2005). However, the optimal duration of treatment continuation remains unclear (Baldwin et al., 2014).

Two previous pairwise meta-analyses of antidepressant treatment for individuals with OCD in the maintenance phase were conducted, both of which included only double-blind randomized placebo-controlled trials (DBRPCTs) with an enrichment design (Batelaan et al., 2017; Donovan, Glue, Kolluri, & Emir, 2010). In these trials, individuals with OCD who had been stabilized on an antidepressant during an open-label phase were subsequently randomized to continue the same antidepressant or switch to a placebo. Both meta-analyses revealed that the antidepressant maintenance group demonstrated a significantly lower relapse rate than the antidepressant discontinuation group (Batelaan et al., 2017; Donovan et al., 2010). However, these meta-analyses did not examine OCD symptom improvement, all-cause discontinuation (as a measure of acceptability), or discontinuation due to adverse events (as a measure of tolerability). Therefore, we conducted an updated systematic review and meta-analysis to address these limitations. In addition, the temporal trajectory of effect sizes for antidepressant maintenance in preventing relapse among individuals with OCD remains unclear. Clarifying this evidence is crucial for determining the optimal duration of maintenance treatment. To address this, the present pairwise meta-analysis compared relapse rates at matched observation time points (i.e. 4, 8, 12, 16, 20, and 24 weeks) between antidepressant discontinuation and maintenance groups, aiming to more precisely characterize the temporal pattern of relapse risk.

## Materials and methods

This systematic review and meta-analysis was conducted in accordance with the Preferred Reporting Items for Systematic Reviews and Meta-Analyses statement (Supplementary Table S1; Page et al., 2021). The study was registered with the Open Science Framework (https://osf.io/mkr34). At least two authors (T.K., K.S., M.H., S.H., and Y.N.) simultaneously and independently performed the literature search and data extraction, and the obtained data were entered into a spreadsheet for analysis. All data were double-checked for accuracy.

### Search strategy and inclusion criteria

The Patient, Intervention, Comparison, and Outcome strategy was used for a formal systematic literature review.

Patient: Individuals with OCD who were stabilized with antidepressants.

Intervention: Antidepressant.

Comparison: Placebo.

Outcomes: Relapse rate at the study endpoint of each DBRPCT (primary outcome); relapse rates in individuals with OCD at 4, 8, 12, 16, 20, and 24 weeks; improvement in OCD symptoms measured using the Yale–Brown Obsessive-Compulsive Scale (Y-BOCS; Goodman et al., 1989) or the Children’s Y-BOCS (Scahill et al., 1997); all-cause discontinuation; and adverse event–related discontinuation.

The authors searched for trials published before May 22, 2025, in the databases of Embase, PubMed, and the Cochrane Central Register of Controlled Trials. The following search terms were used in PubMed and the Cochrane Central Register of Controlled Trials: (Obsessive-Compulsive Disorder [MeSH]) AND (recur* OR relapse) AND (randomized). The following search terms were used in Embase: (‘obsessive compulsive disorder’/exp OR ‘obsessive compulsive disorder’) AND (‘randomized controlled trial (topic)’/exp OR ‘randomized controlled trial (topic)’) AND (‘relapse’/exp OR ‘relapse’). The literature search was conducted without any language restrictions. This study included only DBRPCTs with an enrichment design in which individuals with OCD were stabilized with the antidepressant during the open-label phase and then randomized to receive either the same antidepressant or a placebo. The retrieved trials were assessed against the inclusion and exclusion criteria, and eligible trials were selected. Additional relevant published and unpublished trials, including conference abstracts, were manually searched in the reference lists of the included trials and review articles. Furthermore, clinical trial registries (e.g. ClinicalTrials.gov [http://clinicaltrials.gov/] and the World Health Organization International Clinical Trials Registry Platform [http://www.who.int/ictrp/search/en/]) were searched to ensure comprehensive coverage of eligible trials and to minimize the risk of publication bias. A consensus was reached among the authors to resolve any discrepancies regarding trial selection.

### Data synthesis and extraction

Table 1 shows the definitions of relapse for each study. The authors independently extracted data from all included studies. All analyses were conducted according to the intention-to-treat or modified intention-to-treat principles. Missing data in published systematic review articles were searched when the data required for this study were incomplete. We measured relapse rates from the curves using a ruler to match the observational time points of relapse rates in each DBRPCT for studies that reported Kaplan–Meier survival curves.

**Table 1.** Characteristics of double-blind, randomized, placebo-controlled trials included in our systematic review

Study name (county)	Study duration	Participants	Comorbidities (%)	AD (mg/day, dosage)	AD (mg/day) discontinuation	Concomitant medication	Use of PsT before the trial	Use of PsT during the trial	Age (years)	Male (%)	Caucasian (%)	The definition of relapse
Fineberg et al. (2007) (international)	24 weeks	OP with OCD (DSM-IV-TR) who had responded to 16 weeks prior ESC treatment (≥25% decrease from baseline in Y-BOCS)	None	ESC 10 or 20 (fixed)	ESC 10 for 1 week, then placebo	ZAL, ZOL, ZOP	None	None	35.60 ± 11.76	50.63	78.44	≥5 points increase in Y-BOCS, or lack of efficacy
Foa et al. (2022) (USA)	24 weeks	OP with OCD (DSM–5) who had responded to prior SRI treatment and 25 sessions of ERP therapy (Y-BOCS≤14)	DeD (14.85), AnD (17.82), other (7.92)	Various SRI^a^ (flexible)	SRI dose reduced by 25% weekly and switched to PLA	BEN, STI	25 sessions of ERP therapy	Monthly ERP booster sessions	31.0 ± 11.2	45.54	85.15	NR^c^
Geller et al. (2003) (USA)	16 weeks	PT with OCD (DSM-IV) who had responded to 16 weeks prior PAR treatment (≥25% decrease from baseline in CY-BOCS and CGI-I ≤ 2)	None	PAR 10–60 (flexible)	NR	None	None	None	11.70 ± 2.72	54.40	91.19	≥1 increase in CGI-I for 2 consecutive visits, ≥2 increase in CGI-I at any visit, or CGI-I ≥ 5 at any visit
GSK (1994) (MY–1053) (USA)	52 weeks	OP with OCD (DSM-III-R) who had responded to 26 weeks prior PAR treatment (≥25% decrease from baseline in Y-BOCS and ≥ 2 points decrease in CGI-S)	None	PAR 20–60 (flexible)	Abruptly switched to PLA	CH	None	None	40.92 ± 11.50	59.09	93.18	Return of Y-BOCS to baseline or ≥ 1 point increase in CGI-S
Hollander et al. (2003) (USA)	26 weeks	OP with OCD (DSM-III-R) who had responded to 26 weeks prior PAR treatment (≥25% decrease from baseline in Y-BOCS and ≥ 2 points decrease in CGI-S)	None	PAR 10, 20, 30, 40, 50, or 60 (mean dose = 52.5, fixed)	Abruptly switched to PLA	CH	None	None	42.62 ± 12.56	66.67	97.14	Return of Y-BOCS to baseline or ≥1 point increase in CGI-S
Koran et al. (2002) (USA)	28 weeks	OP with OCD (DSM-III-R) who had responded to 52 weeks prior SER treatment (≥25% decrease from baseline in Y-BOCS and CGI-I ≤ 3)	None	SER 50–200 (flexible)	SER decreased by 50 every 3 days, substituted with PLA	CH	None	None	39.35 ± 11.12	55.61	NR	≥5 points increase in Y-BOCS, Y-BOCS ≥ 20, or ≥1 points increase in CGI-I
Koran et al. (2005) (USA)	7 weeks	OP with OCD (DSM-IV) who had responded to 12 weeks prior MIR treatment (>25% decrease from baseline in Y-BOCS)	DeD (NR), AnD (NR)	MIR 30–60 (flexible)	MIR decreased by 15 every 3–4 days, substituted with PLA	None	None	None	34.5 ± 10.24^b^	50.00^b^	NR	≥25% increase from baseline in Y-BOCS or CGI-I ≥ 6
Romano et al. (2001) (USA)	52 weeks	OP with OCD (DSM-IV) who had responded to 16 weeks prior FLUO treatment (≥25% decrease from baseline in Y-BOCS or CGI-I ≤ 2)	None	FLUO 20–60 (flexible)	NR	None	None	None	40.81 ± 13.04	42.86	94.29	≥50% loss in Y-BOCS improvement, Y-BOCS≥19, CGI-I ≥ 6
NCT00215137 (2009) (USA)	8 weeks	PT with OCD who had responded to 8 weeks prior ESC treatment (CGI-I ≤ 2)	None	ESC 10 or 20 (flexible)	NR	None	None	None	39.14 ± 13.27^b^	35.71^b^	NR	NR

*Note:* AD, antidepressant; AnD, anxiety disorder; BEN, benzodiazepines; CH, chloral hydrate; CGI-I, Clinical Global Impression-Improvement score; CGI-S, Clinical Global Impressions-Severity of Illness Scale score; CY-BOCS, Children’s Yale-Brown Obsessive Compulsive Scale total score; DeD, depressive disorder; DSM-TR, Diagnostic and Statistical Manual of Mental Disorders-Text Revision; ESC, escitalopram; ERP, exposure/response prevention; FLUO, fluoxetine; FLUV, fluvoxamine; MIR, mirtazapine; NR, not report; OCD, obsessive-compulsive disorder; OP, outpatient; PAR, paroxetine; PT, patient; PsT, psychotherapy; SER, sertraline; SRI, serotonin reuptake inhibitor; STI, stimulants; USA, United States of America; Y-BOCS, Yale-Brown Obsessive Compulsive Scale total score; ZAL, zaleplon; ZOL, zolpidem; ZOP, zopiclone.

a Mean dose (mg/day): citalopram 30.0, clomipramine 150.0, escitalopram 27.6, fluoxetine 53.5, fluvoxamine 228.3, PAR paroxetine, and sertraline 166.0.

b Data were collected at baseline during the prerandomization phase of the study.

c Although the study by Foa et al. did not report relapse rates, we treated the outcome ‘removal due to clinical worsening’ as a proxy for relapse.

### Meta-analysis methods

This pairwise meta-analysis utilized a random-effects model (DerSimonian & Laird, 1986). Risk ratios (RRs) for dichotomous data and standardized mean differences (SMDs) for continuous data were calculated, along with their respective 95% confidence intervals (CIs). The *I*^2^ statistic was used to assess heterogeneity among the included studies, with an *I*^2^ of ≥50% indicating considerable heterogeneity (Higgins et al., 2022). Furthermore, a single-group summary meta-analysis was conducted to identify the exact relapse rates with 95% CIs in both the maintenance and discontinuation groups. The absolute risk reduction (ARR) and the number needed to treat to benefit (NNTB) for relapse rates were also estimated when the pairwise meta-analysis revealed significant differences between the treatment groups.

We conducted two sensitivity analyses of relapse rates at the study endpoint of each DBRPCT: one excluding a study involving children and adolescents (Geller et al., 2003) and another excluding a study that included participants who received exposure and response prevention (ERP) therapy (Foa et al., 2022). Additionally, given that the meta-analysis of relapse rates at 16 weeks exhibited considerable heterogeneity, we performed the same sensitivity analyses for this outcome. Moreover, a meta-regression analysis for the primary outcome was conducted to investigate confounding factors (Supplementary Table S2). Informed by recent clinical trial literature (Foa et al., 2022), the following potentially confounding factors were considered: method of antidepressant discontinuation (abrupt versus gradual), serotonin reuptake inhibitor (SRI) type based on half-life (≤26 versus >26 hours), presence or absence of comorbidities, and study duration (weeks). Publication year was also included as a moderator, as an increase in placebo response has been observed in acute-phase studies, particularly in recent decades (Kotzalidis et al., 2019). Egger’s test was used to identify potential publication bias, as funnel plots with <10 studies are not meaningful (Higgins et al., 2022). Comprehensive Meta-Analysis software version 3 (Biostat Inc., Englewood, NJ, USA) was used for all statistical analyses. We assessed the risk of bias using the Cochrane Risk-of-Bias Tool for Randomized Trials, version 2 (Higgins et al., 2022).

## Results

Supplementary Figure S1 illustrates the literature search and selection strategy. Initially, 323 articles were identified, of which 46 were duplicates. After title and abstract screening, 271 articles were excluded. Three additional articles were identified through manual searching (Foa et al., 2022; GSK, 1994; Koran, Gamel, Choung, Smith, & Aboujaoude, 2005). Finally, we identified nine DBRPCTs involving a total of 1084 individuals with OCD (53.3% male; mean age: 32.8 years; Fineberg, Tonnoir, Lemming, & Stein, 2007; Foa et al., 2022; Geller et al., 2003; GSK, 1994; Hollander et al., 2003; Koran et al., 2005; Koran, Hackett, Rubin, Wolkow, & Robinson, 2002; NCT00215137, 2009; Romano, Goodman, Tamura, & Gonzales, 2001). Table 1 summarizes the characteristics of the included DBRPCTs. Although one study included participants who were receiving various SRIs, such as citalopram and clomipramine (Foa et al., 2022), the other studies included only participants receiving a single, specific antidepressant: escitalopram, fluoxetine, mirtazapine, paroxetine, or sertraline. One study included participants who received ERP therapy (Foa et al., 2022), whereas the other studies did not include participants who received psychotherapy, such as behavioral therapy. The mean study duration was 26.3 weeks. No studies demonstrated a high risk of bias in any domain of the risk-of-bias 2 tool (Supplementary Figure S2).

The antidepressant maintenance group had lower relapse rates at the study endpoint of each DBRPCT (RR [95% CI] = 0.53 [0.42–0.68], *I*^2^ = 31.7%, ARR = 21.0%, NNTB = 5), lower all-cause discontinuation rates (RR [95% CI] = 0.70 [0.54–0.90]), and lower adverse event-related discontinuation rates (RR [95% CI] = 0.46 [0.24–0.88]) compared with the antidepressant discontinuation group (Table 2 and Supplementary Figure S3). Stated differently, the placebo group demonstrated significantly higher rates of relapse, all-cause discontinuation, and adverse event-related discontinuation than the antidepressant group. Moreover, the antidepressant maintenance group outperformed the antidepressant discontinuation group in terms of OCD symptom improvement (SMD [95% CI] = −0.27 [−0.53 to −0.02]; Table 2 and Supplementary Figure S3). Egger’s test revealed no publication bias for the primary outcome (*p* = 0.31).

**Table 2.** Results of meta-analysis

Dichotomous variables	K (n)	RR (95% CI)	*p* value	*I*^2^ (%)
Relapse rate at the study endpoint of each DBRPCT	8 (1065)	0.53 (0.42–0.68)	<0.01	31.7
All-cause discontinuation	9 (1084)	0.70 (0.54–0.90)	<0.01	67.8
Adverse event-related discontinuation	6 (924)	0.46 (0.24–0.88)	0.02	25.5

Continuous variable	K (n)	SMD (95% CI)	*p* value	*I*^2^ (%)
Improvement in OCD symptoms^a^	8 (951)	−0.27 (−0.53, −0.02)	0.03	63.9

*Note*: 95% CI, 95% confidence interval; DBRPCT, double-blind randomized placebo-controlled trial; OCD, obsessive-compulsive disorder; RR, risk ratio; SMD, standardized mean difference.

a OCD symptoms were assessed using the Yale–Brown Obsessive Compulsive Scale (Goodman et al., 1989) or the Children’s Yale–Brown Obsessive Compulsive Scale (Scahill et al., 1997).

**Figure 1.**
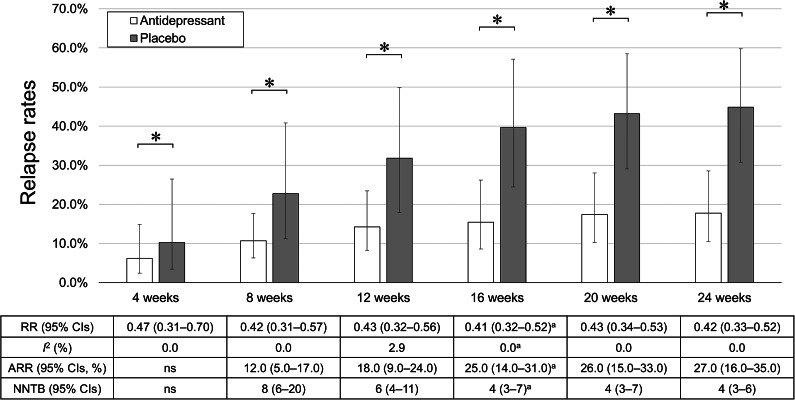
Relapse rates. ^a^ Although the primary meta-analysis demonstrated that the maintenance group had a lower relapse rate at 16 weeks compared with the discontinuation group (RR [95% CI] = 0.47 [0.34–0.66]), the result exhibited considerable heterogeneity (*I*^2^ = 57.6%). In contrast, the sensitivity analysis excluding the study involving children and adolescents also showed lower relapse rates in the maintenance group at 16 weeks but without considerable heterogeneity (*I*^2^ = 0.0%). Therefore, we present the results of this sensitivity analysis in the figure. *Note*: ARR, absolute risk reduction; CIs, confidence intervals; NNTB, number needed to treat to benefit; ns, not significant; RR, risk ratio. *p < 0.05.

The antidepressant maintenance group demonstrated lower relapse rates at 4, 8, 12, 16, 20, and 24 weeks compared with the antidepressant discontinuation group (Figure 1 and Supplementary Figure S3). The results for relapse rates at all time points, except 16 weeks, did not exhibit considerable heterogeneity (Supplementary Figure S3).

For the sensitivity analysis of the primary outcome, results consistent with the primary meta-analysis were observed both when excluding the study involving children and adolescents (RR [95% CI] = 0.48 [0.39–0.59], *I*^2^ = 0.0%) and when excluding the study that included participants who received ERP therapy (RR [95% CI] = 0.53 [0.40–0.70], *I*^2^ = 41.4%; Figure 1 and Supplementary Figure S3). The primary meta-analysis demonstrated that the maintenance group had a lower relapse rate at 16 weeks compared with the discontinuation group (RR [95% CI] = 0.47 [0.34–0.66]). Similar findings were observed in the two sensitivity analyses: one excluding the study involving children and adolescents (RR [95% CI] = 0.41 [0.32–0.52]) and the other excluding the study including participants who received ERP therapy (RR [95% CI] = 0.46 [0.31–0.68]). Both the primary analysis (*I*^2^ = 57.6%) and the sensitivity analysis excluding the ERP therapy study (*I*^2^ = 65.9%) exhibited considerable heterogeneity, whereas the sensitivity analysis excluding the child and adolescent study did not (*I*^2^ = 0.0%). No moderators were found to be significantly associated with the magnitude of the effect size for the primary outcome (Supplementary Table S2).

## Discussion

Our meta-analysis revealed comparable RRs for relapse rates at 4, 8, 12, 16, 20, and 24 weeks; however, the ARR slightly increased over time, indicating a corresponding slight decrease in the NNTB (Figure 1). The average relapse rates in both the maintenance and discontinuation groups increased over time, but the relapse rate increased earlier and more substantially in the discontinuation group than in the maintenance group (Figure 1). These results indicate that in individuals with OCD whose acute symptoms improve with antidepressant treatment, maintenance treatment should be continued for at least 24 weeks to prevent relapse. Moreover, our findings indicate that individuals with OCD may benefit from continuing antidepressant treatment beyond 24 weeks, given the changes in 24-week relapse rates observed over time in our meta-analysis and the substantial preventive effect of antidepressants that appears to persist beyond this period. However, clinicians should be mindful of adverse events associated with antidepressants, as the safety profiles of these medications vary widely (Kishi et al., 2024).

Our meta-analysis revealed that the antidepressant maintenance group had a significantly lower all-cause discontinuation rate compared with the discontinuation group. All-cause discontinuation is a critical outcome in DBRPCTs of pharmacological treatments for mental illnesses, as it reflects efficacy, tolerability, and other factors (Kishi et al., 2021, 2022; Kishi, Ikuta, et al., 2023; Kishi, Matsuda, Sakuma, Okuya, & Iwata, 2020; Kishi, Sakuma, et al., 2023). Several major reasons for discontinuation in such trials were identified, including lack of efficacy (as determined by the patient and/or physician), adverse events, withdrawal of consent, loss to follow-up, and protocol violations. Therefore, all-cause discontinuation serves as a comprehensive measure that reflects multiple aspects of treatment administration and feasibility in clinical practice (Lieberman et al., 2005). Thus, antidepressants are considered to have good acceptability for individuals with OCD during the maintenance phase. Furthermore, our meta-analysis revealed that the antidepressant maintenance group had a lower rate of adverse event–related discontinuation compared with a placebo. Because adverse event–related discontinuation may include patients who discontinued due to disease worsening, we were unable to interpret these results in greater depth.

However, despite continued antidepressant treatment, approximately 18% of individuals with OCD experienced relapse within 24 weeks. Robust evidence supports the efficacy and favorable safety profile of non-pharmacological interventions, such as psychotherapy and neuromodulation therapy, for the treatment of OCD (Dehghani-Arani et al., 2024; Fineberg et al., 2023; Harmelech, Roth, & Tendler, 2023; Hirschtritt et al., 2017; Pellegrini et al., 2022; Stein et al., 2019; Suhas et al., 2023; Vicheva, Osborne, Krieg, Ahmadi, & Shotbolt, 2025). The study by Foa et al. (2022), which included participants who received ERP therapy, reported that changes in scores on the Y-BOCS, Hamilton Depression Rating Scale (Hamilton, 1967), and the Quality-of-Life Enjoyment and Satisfaction Questionnaire–Short Form (Endicott, Nee, Harrison, & Blumenthal, 1993) were comparable between the SRI discontinuation and continuation groups. However, because participants who tapered their SRI experienced higher rates of clinical worsening, the authors emphasized that tapering may require careful monitoring. Therefore, future research should investigate the combination of treatments that most effectively prevent relapse in individuals with OCD (Reid et al., 2025).

Our study had several limitations. First, a previous network meta-analysis revealed that the effect sizes of OCD symptom improvement differed among antidepressants (Skapinakis et al., 2016). However, our study did not evaluate differences in efficacy and safety profiles among individual antidepressants for the maintenance treatment of OCD. Moreover, our meta-analysis did not include studies that focused exclusively on older antidepressants, such as tricyclic antidepressants. Second, the number of studies and participants was small. Only one study focused on children and adolescents with OCD in the maintenance phase. Although the current meta-regression analysis examined whether the type of SRI based on half-life and the method of antidepressant discontinuation were associated with the magnitude of the primary outcome, a number of studies included in meta-regression analyses were also small, limiting the robustness of the results. Third, all of the studies included in this meta-analysis, except the study by Foa et al. (2022), were relatively old, having been conducted before 2009. Fourth, important clinical issues regarding treatment decision-making in routine clinical practice (e.g. monotherapy or combination of antidepressants with nonpharmacological treatments) were not addressed. Sixth, data on relapse rates at time points beyond 24 weeks were insufficient; thus, we were unable to conduct a meta-analysis for those outcomes. Therefore, at this time, the necessity of a longer period of antidepressant treatment for these individuals remains unclear.

In conclusion, our findings indicate that individuals with OCD may benefit from continued antidepressant treatment, provided that it is well tolerated.

## Supporting information

Kishi et al. supplementary material Kishi et al. supplementary material

## Data Availability

The data used for the current study were reported in the articles of the studies included in our meta-analysis.

## Supplementary material

The supplementary material for this article can be found at http://doi.org/10.1017/S0033291725101578.
